# Evolution of apoptosis-like programmed cell death in unicellular protozoan parasites

**DOI:** 10.1186/1756-3305-4-44

**Published:** 2011-03-25

**Authors:** Szymon Kaczanowski, Mohammed Sajid, Sarah E Reece

**Affiliations:** 1 Institute of Biochemistry and Biophysics, Polish Academy of Sciences, Warszawa Pawinskiego 5A 02-106, Poland; 2 Leiden Malaria Research Group, Leiden University Medical Centre, Department of Parasitology, Albinusdreef 2, Kamer P4-35, 2333 ZA, Leiden, Netherlands; 3 Centre for Immunity, Infection and Evolution, Institutes of Evolution, Infection and Immunity, School of Biological Sciences, University of Edinburgh, Edinburgh EH9 3JT, UK

## Abstract

Apoptosis-like programmed cell death (PCD) has recently been described in multiple taxa of unicellular protists, including the protozoan parasites *Plasmodium, Trypanosoma *and *Leishmania*. Apoptosis-like PCD in protozoan parasites shares a number of morphological features with programmed cell death in multicellular organisms. However, both the evolutionary explanations and mechanisms involved in parasite PCD are poorly understood. Explaining why unicellular organisms appear to undergo 'suicide' is a challenge for evolutionary biology and uncovering death executors and pathways is a challenge for molecular and cell biology. Bioinformatics has the potential to integrate these approaches by revealing homologies in the PCD machinery of diverse taxa and evaluating their evolutionary trajectories. As the molecular mechanisms of apoptosis in model organisms are well characterised, and recent data suggest similar mechanisms operate in protozoan parasites, key questions can now be addressed. These questions include: which elements of apoptosis machinery appear to be shared between protozoan parasites and multicellular taxa and, have these mechanisms arisen through convergent or divergent evolution? We use bioinformatics to address these questions and our analyses suggest that apoptosis mechanisms in protozoan parasites and other taxa have diverged during their evolution, that some apoptosis factors are shared across taxa whilst others have been replaced by proteins with similar biochemical activities.

## Introduction

Apoptosis-like programmed cell death (PCD) has been described in multiple taxa of unicellular protists, including the protozoan parasites *Plasmodium *[1,2], *Trypanosoma *[3,4] and *Leishmania *[5]. PCD in protists appears to share some morphological features with apoptosis in multicellular organisms, including chromosomal condensation, nuclear DNA fragmentation, cell shrinkage, loss of mitochondrial membrane potential, formation of apoptotic bodies, and the externalisation of phosphatidylserine [2,4]. However, without knowledge of the molecular mechanisms involved in the apoptosis-like PCD of parasites, it is unclear which markers are expected to be observed and under which conditions. Apoptosis in multicellular organisms is initiated in response to a wide variety of stress factors, ranging from cell senescence to oxidative damage [6,7], and is executed by the activation of the caspase family of cysteine proteases [8]. Although apoptosis-like PCD in unicellular organisms can also be initiated by a variety of stresses [4,5,9] and the morphological features (diagnostics) are similar across multicellular and unicellular taxa [2,4,9], much of the molecular machinery of unicellular organisms appears to differ. For example, canonical caspases are encoded only in the *Metazoan *genomes [10-12].

This mixture of similarities in the basic features of apoptosis but key differences in the underlying mechanisms across taxa has resulted in controversy over whether apoptosis, or a form thereof, is actually the process being observed in protozoan parasites. This controversy must be resolved as the possibility of manipulating cell death pathways in parasites may offer a new avenue for disease control. Empirical tests of gene function have made progress in identifying some the molecules involved in executing apoptosis, and bioinformatics offers a complimentary approach to integrate these results across taxa. Bioinformatic comparisons of multicellular model systems, protozoan parasites, and their free-living relatives can reveal candidate genes that encode proteins with similar function across taxa with different modes of life, and reveal the extent of conservation or divergence in their sequences. Here, we use a bioinformatics approach to identify initiators and executioners of cell death that appear to be shared between protozoan parasites and multicellular taxa. This type of analysis can aid functional studies in the search for possible drug targets and can also shed light on whether these shared mechanisms have arisen through convergent or divergent evolution, which is necessary to inform studies asking 'why' such traits have evolved.

Understanding the evolutionary and ecological pressures shaping the expression of apoptosis-like PCD in protozoan parasites is also central to the success of interventions targeting this trait. The evolution and ecology of apoptosis-like PCD in protozoan parasites is addressed in Pollitt *et al*. [13], but briefly: natural selection is predicted to favour genotypes (clones) in which some parasites undergo apoptosis if it increases the transmission of their clone-mates (kin). This hypothesis predicts that parasites should employ apoptosis according to their relatedness and in line with changes in their density. For example, a parasite genotype (a group produced through clonal expansion) may benefit from reducing its proliferation rate if uncontrolled replication is likely to result in premature death of its host or vector. In a situation when an infection is composed of close kin (i.e. clone-mates), the parasites that die may facilitate the transmission (fitness) of their relatives and indirectly pass on shared genetic information by being prudent. However, if an infection contains multiple, unrelated, co-infecting genotypes, then the benefits of PCD are shared across all genotypes. In this situation, if parasites were to undergo PCD they would be helping unrelated competitors - which is not a strategy favoured by natural selection.

Recent empirical work suggests that *Plasmodium *parasites, like bacteria, are capable of coordinating behaviours as a clonal group and alter their reproductive strategies in response to changes in both relatedness and density [13-17]. If the expression of apoptosis-like PCD is influenced by relatedness and density then identifying the mechanisms underpinning this trait could also reveal how parasites co-ordinate their social behaviour. However, testing whether apoptosis-like death in single-celled organisms *per se *is challenging and to date, few studies provide supportive data (reviewed in [18]). Alternatively, apoptosis in single-celled organisms could potentially be an unfortunate but unavoidable consequence of some cellular processes (such as ageing [18]). In this case, identifying the molecules involved in apoptosis- like PCD could reveal genes associated in pleiotropic interactions. As the same selection pressures may not necessarily be involved in both the evolution and maintenance of apoptosis-like death, within and across parasite taxa, the identification of machinery involved in the initiation and execution of death is required.

### Apoptosis in multicellular organisms

During apoptosis in multicellular organisms, the cell activates suicide machinery that culminates in chromosomal condensation and nuclear DNA fragmentation [19,20]. In mammals, the intrinsic apoptosis pathway is activated by permeabilisation of the mitochondrial membrane [21,22] and release of the mitochondrial proteins, cytochrome *c *[23], apoptosis induction factor (AIF) [24] and endonuclease G (EndoG) into the cytoplasm [25]; activation of the cysteine endoprotease family of caspases is key in the catabolic execution of apoptosis [8]. Cytochrome *c*, AIF and caspases activate nucleases. In plants, apoptosis is also activated by the release of factors from the mitochondria (though cytochrome *c *seems not to be involved), which results in chromatin condensation and DNA degradation [26]. Plants do not contain canonical caspases, but their orthologues in the metacaspase family fulfil this role. Interestingly, plant metacaspases have different substrate specificities compared to mammalian caspases but they still exhibit a biological activity akin to caspases that results in apoptosis [26,27].

### Apoptosis like PCD in unicellular organisms

Physical and chemical stresses have been shown to initiate apoptosis in a variety of free living unicellular organisms, such as the yeast *Saccharomyces cerevisiae *[9] and the freshwater algae, *Chlamydomonas reinhardtii *[28]. Many of the elements of the apoptosis network of yeast are similar to those described previously in mammals [29,30]. This includes translocation of the mitochondrial proteins AIF [31], EndoG [32] and cytochrome *c *[33] to the cytoplasm, where their modes of action resemble those of their mammalian counterparts. Like plants, yeast cells express the caspase-homologues, metacaspases [10,11]. The disruption of yeast metacaspases abrogates hydrogen peroxide-induced apoptosis, whereas over-expression of metacaspases increases hydrogen peroxide-induced proteolytic activity of metacaspase, resulting in apoptosis [10]. Apoptosis also occurs in free-living unicellular ciliated protists during conjugation. For example, in *Tetrahymena thermophila*, the parental macronucleus is selectively eliminated from the cytoplasm by an apoptosis-like process [34].

### Apoptosis like PCD in parasites

Compared to apoptosis in model organisms, understanding of PCD mechanisms in parasitic organisms remains limited. However, studies have revealed apoptosis markers in diverse parasite taxa, including *Trypanosomes *[3,4], *Leishmania *[5], and *Plasmodium *[1,2,35,36]. Typical features of apoptosis-like cell death are observed in these organisms, such as cell shrinkage, nuclear condensation or DNA fragmentation, activation of caspase-family proteases and accumulation of cytochrome *c *in the cytoplasm [2,4]. Apoptosis can be induced using a variety of agents, such as drugs or oxidative stress resulting from hydrogen peroxide [4,5] or naturally occurring nitric oxide donors [35]. Rates of apoptosis have been quantified for *Plasmodium *parasites [1,2,13] - in *P. berghei *up to 50% of parasites in the mosquito midgut (ookinetes) undergo apoptosis [1]. However, there is considerable variation in the timing and proportion of ookinetes positive for apoptosis, both across species and according to the markers used [13]. The functions of just two apoptosis factors of unicellular protozoans, AIF of *Tetrahymena *[37] and EndoG of trypanosomatids [38], have been experimentally confirmed. Whilst such studies offer an elegant way to experimentally determine the functions of gene products, this can be complicated if molecules are involved in multiple regulatory pathways or are only active under certain situations. Furthermore, the complexity of apoptosis induction and execution mechanisms in other taxa, may suggest that multiple pathways are also involved in protozoa.

### Apoptosis executors: caspases

Biochemical experiments suggest that proteases are activated during apoptosis in *Plasmodium *[2] and trypanosomatid parasites of the genus *Leishmania *[39]. However, it is not clear which proteases are central to the execution of apoptosis-like cell death. Although the genomes of these parasites do not encode 'classical' caspases, they encode the close family members, metacaspases [40,41], as do other non-mammalian eukaryotes [10,11,26,27]; the metacapases, like the true caspases, belong to family C14 with the clan CD cysteine proteases [42]. It has been suggested that metacaspases can carry out a functionally analogous biological roles to metazoan caspases, and although data are scarce, they have been linked to programmed cell death in plants and yeast [10,11,26,27]. Whether metacaspases are directly involved in protozoan apoptosis remains unclear as evidence for their involvement is controversial, for the reasons outlined below.

First, whilst vertebrate caspases have specificity for Asp and P1, to date, no characterised cysteine protease from protozoan parasites has the same P1 preference; indeed, where studied, metacaspases, including the plant metacaspases, have an Arg and/or Lys specificity at P1. Secondly, *Trypanosoma brucei *possesses five metacaspase genes. Two of them (MCA1 and MCA2) lack a predicted active site cysteine and so are probably not active cysteine proteases [43]. A triple null mutant lacking the active metacaspases was isolated after sequential gene deletions but activation of apoptosis programs was still observed in this mutant. These experiments demonstrate that metacaspases are not solely involved in the apoptosis-like cell death of trypanosomatids [44]. Thirdly, *Plasmodium *parasites possess three metacaspases. Addition of the caspase inhibitor Z-VAD-fmk to *Plasmodium berghei *results in a reduction of apoptotic ookinetes, and also an increase in the number of oocysts in the mosquito midgut [1]. However, the use of probes designed for mammalian cells, such as Z-VAD-fmk, at high concentrations could result in the inhibition of cysteine proteases other than metacaspases. Such an off- target effect could be further compounded by the small hydrophobic residue, Ala, at P2 of Z-VAD-fmk as it has a preference for papain family cysteine proteases [45]. Studies of *Plasmodium berghei *metacaspase 1 reveal that this protein is expressed in female gametocytes and in all mosquito stages, including the midgut stages. However, when PbMC1 was deleted the authors failed to detect a phenotype related to apoptosis [46] though they did not observe apoptosis-like cell death in their wild-type control line either. The picture is further complicated by the suggestion that MC1 deletion is involved in elevating parasite density, but not *via *prevention of apoptosis [13].

In general, knowledge of evolutionarily conserved substrates of caspase-family proteases involved in apoptosis is still very limited. In fact, TSN (Tudor Staphylococcal Nuclease) a nuclease involved in the process of RNA splicing, is the only well-known substrate of apoptosis proteases of higher plants and mammals [27]. Caspase-mediated proteolysis of TSN is important for the progress of apoptosis because inactivation of TSN expression in plant [27] and human cells [27] leads to the induction of the apoptosis program. Cleavage of TSN inhibits its activation of mRNA splicing and its ribonuclease activity and this is an important role in the execution of apoptosis. Identifying the substrates of protozoan metacapases would provide another approach for functional studies to assess the role of metacaspases in apoptosis. To address this, we used BLASTP [47] to detect homologues of TSN in the genomes of protozoan parasites and found homologues of this protein across trypanosomatids and Apicomplexa (e-values < 5.1e-54). Specifically, we recovered the following putative TSN nucleases (top blast hits) in: *L. major *(uniprot ID Q4Q5I7_LEIMA), *T. cruzii *(uniprot ID Q4DY53_TRYCR), *P. falciparum *(plasmodb PF11_0374), and *T. gondii *(uniprot ID B6KG97_TOXGO). We suggest that as TSN proteins are substrates of animal caspases and plant metacaspases, they may be also substrates of apoptosis proteases in protozoan parasites.

### Apoptosis executors: nucleases

As previously mentioned, nucleosomal fragmentation of DNA is classical hallmark of apoptosis in metazoan systems [19,20]. DNA fragmentation is caused by apoptosis nucleases, which cut nuclear DNA during the apoptosis program. The putative primary target of DNases is parasitic plasmids and viruses of bacteria. This hypothesis is supported by the fact that close homologous proteins of nucleases (for example EndoG [25,32,38], ZEN1 [48], NUC1 [49]) are present in bacterial genomes. Many types of DNases involved in apoptosis have been identified in different species. Table 1 presents the results of our BLAST homology searches for homologues of different apoptosis nucleases, which are discussed below.

**Table 1:** BLAST homology searches using apoptosis DNases as queries

Name Species ID	CAD	ICAD	EndoG	ZEN1	NUC1
	*D. melanogaster *Q9NDR2	*H. sapiens *DFFA_HUMAN	*H. sapiens *NUCG_HUMAN	*H. vulgare *O81958	*C. elegans *NUC1_CAEEL
*D. melanogaster*	***Q9NDR2 1.5e-236***	Q6NR36 **0.051**	Q7JXB9 **1.4e-61**	**No**	Q7JYM9 **3.2e-46**

*H. sapiens*	O76075 **5.3e-22**	***DFFA_HUMAN 2.1e-161***	***NUGG_HUMAN 1.5e-129***	**No**	A0AUY7 **1.5e-46**

*C. elegans*	**No**	**No**	NUCG_CAEEL **9.2e-56**	**No**	***NUC1_CAEEL 1.0e-198***

*S. cerevisiae*	**No**	**No**	NUC1_YEAST **6.1e-43**	**No**	**No**

*T. brucei*	**No**	**No**	Q581C4 **8.2e-20**	Q585P6 **1.2e-06**	**No**

*L. major*	**No**	**No**	Q4QHF4 **1.4e-19**	Q4QGQ3 **1.3e-06**	**No**

*T. gondii*	**No**	**No**	B9PXN1 **3.7e-23**	B6KFB6 **0.087**	**No**

*P. falciparum*	**No**	**No**	**No**	C0H524 **9.9e-05**	**No**

*T. thermophila*	**No**	**No**	**No**	Q236I5 **0.00016**	Q22N03 **2.1e-35**

*Plants*	**No**	**No**	B9P690 ***(P. trichocarpa) *0.00034**	***O81958 (H. vulgare) 3.7e-146***	**No**

*T. vaginalis*	**No**	**No**	**No**	A2E6R1 **2.9e-06**	A2F6V7 **2.2e-41**

Sequences of five different DNases were used as queries to search the UniProt databases (wublast using EBI www server) [47]. Each query DNase represents a different column and homologues detected in other organisms are presented in each row. The UniProt IDs for query and detected sequences are given and the expected values presented below each ID.

One of the best-known apoptosis nuclease is the mammalian caspase-activated DNase, or CAD [50]. CAD is found in a complex with its inhibitor protein, ICAD, also called DNA fragmentation factor 45 [51]. It has been shown that cleavage of this inhibitor by caspases activates apoptotic degradation. As shown in Table 1, homologues of CAD and ICAD are present in only some animal genomes. Another well-described apoptosis nuclease is endonuclease G [25,32,38], a mitochondrial DNase that is released from the mitochondria during apoptosis. Experimental evidence indicates that the activity of this protein is evolutionarily conserved in multicellular organisms, yeast and trypanosomatids [25,32,38]. As shown in Table 1, EndoG is evolutionarily conserved in the majority of eukaryotic systematic groups, including plants and some Apicomplexa. Surprisingly, there are no obvious orthologues in *Plasmodium *species. It is well known that the *Plasmodium *genome is difficult to work with using bioinformatic approaches (see [52]) so we confirmed this result using PFAM domain analysis [53,54]. The endonuclease NS domain (PFAM ID PF01223) is typical for EndoG protein and is not detected in known *Plasmodium *proteomes.

This observation suggests that deletion of EndoG occurred during the evolution of *Plasmodium *parasites from other Apicomplexa, suggesting that *Plasmodium *parasites must have other apoptosis nucleases. Another typical apoptosis DNase in animal taxa is NUC1 [49]. As shown in Table 1, NUC1 is encoded in the genome of *Tetrahymena *but not in protozoan parasites. In addition to EndoG, ZEN1 is a key apoptosis DNase in plants, [48]. As shown in Table 1, homologues of ZEN1 are present in protists but not in animals or fungi. ZEN1 is a putative apoptosis DNase of parasitic unicellular protists. We suggest that this enzyme may be active during apoptosis in *Plasmodium *parasites, which have neither EndoG nor NUC1 homologues. These results also suggest the hypothesis that the apoptosis of protozoan parasites is more similar to apoptosis processes in plants than animals. However, it is also interesting to note that trypanosomatids have homologues of both Zen1 and EndoG.

### Apoptosis induction factor

**Figure 1:**
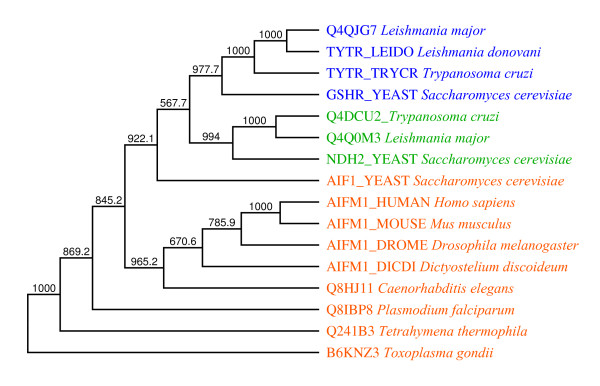
**Consensus phylogenetic parsimony tree of AIF homologues**. Calculated using PHYLIP software with 1000 bootstrap replications. Protein alignment was edited and columns containing gaps were removed. Applied alignments contained 269 informative sites. Red captions indicate orthologues of apoptosis induction factor (AIF), green captions indicate orthologues of NADH dehydrogenase, and blue indicates orthologues of glutathione oxidoreductase. Branch labels indicate bootstrap values. There are no orthologues of AIF in trypanosomatids.

AIF (apoptosis induction factor) is a mitochondrial flavoprotein [24], first characterised in mammalian cells. It has been shown that AIF is sufficient to induce the apoptosis of isolated nuclei and that during apoptosis this protein translocates to the nucleus. Microinjection of AIF into the cytoplasm induces the condensation of chromatin, the digestion of DNA and the dissipation of the mitochondrial transmembrane. It can also function as an electron transferase through a similar mechanism to ferredoxin reductases in bacteria [55]. The pro-apoptosis activity of AIF does not require the cofactor FAD or caspase activity. More recently, apoptotic degradation of DNA induced by AIF has been observed in the nematode *Caenorhabditis elegans *[56] and in the slime mould *Dictyostelium discoideum *[57]. A very recent paper shows [37] that AIF is also an apoptosis induction factor in the free-living ciliate protozoan *Tetrahymena thermophila*. Disruption of AIF delays normal nuclear apoptosis and nuclear condensation. In this case, AIF also translocates from the mitochondria to the macronucleus during nuclear apoptosis. These observations suggest that AIF-mediated programmed cell death is a phylogenetically primitive form of apoptosis conserved in multicellular organisms and protozoa. We used BLAST homology searches to detect homologues of AIF genes in the genomes of parasitic protists. Our phylogenetic analysis, shown in Figure 1, indicates that orthologues of AIF are encoded in the genomes of Apicomplexa but not in the genomes of trypanosomatids. It also indicates that homologues of AIF encoded by genomes of trypanosomatids are more closely related to other proteins than to AIF, suggesting there are no orthologues of AIF in trypanosomatids.

### Apoptosis signalling pathways

In addition to explaining the processes involved in how a cell executes its apoptosis program, it is also important to understand how it is regulated. For example, apoptosis is not always an irreversible process, and apoptosis can interact with other forms of cell death such as autophagy. In mammals, apoptosis can be induced *via *the activation of death signalling complexes [40]. These include ligands (for example TNF), receptors (for example FAS, TNFR1) and adaptors such as TRAF and MATH. The majority of these proteins have no obvious orthologues outside multicellular organisms [see [40] for a review], but detection of evolutionarily relationships between animals and unicellular homologous signalling proteins requires very sensitive tools for domain analysis [40]. However, it is also possible that mechanisms for the regulation of apoptosis are less evolutionarily conserved than the machinery involved in execution of apoptosis. If different circumstances elicit an apoptosis response in different parasite species, then a variety of regulatory mechanisms might be required, but could signal to the same execution machinery.

Another key gap in understanding apoptosis is how cells decide whether or not to initiate programmed death. For example, nutrient sensing pathways [58] are well understood and could provide signals to initiate programmed death *via *autophagy in response to starvation. If apoptosis-like PCD is analogous to suicide, evolutionary theory predicts that it should be conditionally initiated in response to the density and relatedness of parasites within the infection (reviewed in [13]). If so, how do individual parasite cells detect this information and determine if their circumstances merit apoptosis or proliferation? For example, are the stress factors known to elicit apoptosis, actually detected by parasites to gather information about density and relatedness? An obvious parallel here is the regulation of social behaviours in bacteria, through quorum sensing [59]. Clonally related groups of bacteria use quorum sensing to initiate density-dependent behaviours appropriate to their circumstances, including: secretion of antibiotics, production of extracellular matrix and biofilm formation, emission of light, and switches in mode of motility [59]. It is hard to predict if such homologues have similar function in eukaryotes because signaling and regulatory networks evolve rapidly [60-62]. Therefore, whilst many genes involved in quorum-sensing networks have been verified, identifying homologues in the genomes of prokaryotic and eukaryotic organisms is very challenging. Alternatively, recent work revealing receptors involved in density-dependent differentiation of *Trypanosoma brucei *may provide a starting point in the search for density-dependent influences in apoptosis. The transmission of trypanosomes to the tsetse fly requires differentiation from "slender forms" to "stumpy forms" which circulate in the host in G0 arrest, pre-adapted for transmission [63]. The production of stumpy forms is density-dependent and involves a density-dependent signal (SIF), which results in expression of transporters in the PAD family that convey signals for environmental change [63]. However, our BLASTP searches did not identify homologous proteins of these receptors in *Plasmodium *or *Tetrahymena*, supporting the suggestion that there is considerable variation in mechanisms regulating apoptosis-like PCD within protozoan parasites.

### Apoptosis- like PCD without mitochondria

*Trichomonas vaginalis *does not have mitochondria [64] but instead has hydrogenosomes. Hydrogenosomes are reduced both structurally and biochemically compared to the classical mitochondria and recent studies suggest that they share a common ancestry with mitochondria [64]. In *T. vaginalis*, apoptosis-like cell death can be induced by treatment with pro-apoptosis drugs (caspase activators) or nutrient depletion in [65,66], and pancaspase inhibitors are able to prevent apoptosis. DNA fragments in canonical apoptosis have a defined length and are detected with a typical electrophoretic ladder profile. In contrast, *T. vaginalis *exhibits DNA fragmentation without a specific scale pattern and fragments are observed as a smear.

We used bioinformatics to ask whether elements of apoptosis machinery appear to be shared between mitochondrial and amitochondrial protozoan parasites, focussing on AIF, EndoG and cytochrome *c *using BLASTP searches. When baker yeast cytochrome *c *(uniprot ID CY1_YEAST) and human endonuclease G (uniprot ID NUCG_HUMAN) were used as a query sequences the best hits were detected with very low e-values (0.56 and 0.6), suggesting that homologoues of these proteins are not present in *T. vaginalis*. However, when using *Tetrahymena *AIF, six strong hits were detected with e-values smaller than 0.01. We then checked this result using reciprocal blast with our HT-SAS annotation server [67,68]. This reveals that putative AIF homologoues are more closely related with bacterial flavoproteins, thioredoxin reductase, nitric oxide reductase flavorubredoxin and dihydrolipoyl dehydrogenase. We also detected homologues of the apoptosis factors: DNases ZEN1, NUC1 (see Table 1) and TSN (uniprot ID A2DM81_TRIVA; e-value 2.2e-34) when using BLASTP homology searches in the uniprot database. These analyses suggest that mitochondrial apoptosis factors are absent in *T. vaginalis *but that their function has been replaced by nuclear apoptosis factors.

## Conclusions

### Putative mechanism of apoptosis in unicellular protozoa

We suggest the following putative, general, mechanism of apoptosis in most protozoan parasites. Apoptosis like PCD is induced by caspase-family proteases and the execution of apoptosis requires the release of EndoG and AIF from the mitochondria. During apoptosis, cysteine proteases similar to caspases cut molecules, including those similar to TSN. Nucleosomal fragmentation is caused by the apoptosis DNases ZEN1 and EndoG. There are some differences in these mechanisms among different taxa. EndoG has been lost in the *Plasmodium *and ciliate lineages, and AIF has been lost from trypanosomatids. NUC1 is encoded only in the genomes of free-living ciliates and not in those of protozoan parasites. In parasites without mitochondria, such as *T. vaginalis*, mitochondrial apoptosis machinery is replaced by nuclear machinery.

### Apoptosis of unicellular protozoa evolved due to divergent evolution

We suggest that the mechanisms involved in apoptosis of unicellular parasites are due to divergent evolution, but processes and morphologies involved are similar across animals, protists and fungi. For example, the mitochondrial proteins, cytochrome C and EndoG, induce apoptosis in different taxa, and as these proteins are both involved in electron transport. We suggest that their function in apoptosis-like death could be due to co-option because it is unlikely that such a co-option event would independently occur often during the evolution of the same set of proteins, and this may explain why different inducers have evolved in different systematic groups. Another key stage in the execution of apoptosis is fragmentation of nuclear chromatin by apoptosis DNases. We suggest that over the course of evolution, apoptosis enzymes have been replaced by others with similar activities. For example, animals and plants use different apoptosis DNases, and animals use different proteases to fungi and plants. This gene replacement may explain the existence of apoptosis enzymes and mechanisms in unicellular protists that are very different from those in those in other systematic groups. This fact also highlights a limitation of comparative genomics; in using this approach, the detection of divergent genes is possible, but traits and mechanisms that arise through convergent evolution are hard to identify - absence of evidence does not necessarily mean evidence of absence.

## Competing interests

The authors declare that they have no competing interests.

## Authors' contributions

SK collected results presented here and wrote first draft of the manuscript. SR, SK, and MS all participated in the formation of the final version of manuscript.

## References

[B1] Al-Olayan EM Williams G Hurd H Apoptosis in the malaria protozoan, *Plasmodium berghei*: a possible mechanism for limiting intensity of infection in the mosquito Int J Parasitol 2002 32 1133 1143 10.1016/S0020-7519(02)00087-5 12117496

[B2] Arambage SC Grant KM Pardo I Ranford-Cartwright L Hurd H Malaria ookinetes exhibit multiple markers for apoptosis-like programmed cell death *in vitro* Parasit Vectors 2009 2 32 10.1186/1756-3305-2-32 19604379 PMC2720949

[B3] Ameisen JC Idziorek T Billaut-Mulot O Loyens M Tissier JP Potentier A Ouaissi A Apoptosis in a unicellular eukaryote (*Trypanosoma cruzi*): implications for the evolutionary origin and role of programmed cell death in the control of cell proliferation, differentiation and survival Cell Death Differ 1995 2 285 300 17180034

[B4] Duszenko M Figarella K Macleod ET Welburn SC Death of a trypanosome: a selfish altruism Trends Parasitol 2006 22 536 542 10.1016/j.pt.2006.08.010 16942915

[B5] Das M Mukherjee SB Shaha C Hydrogen peroxide induces apoptosis-like death in *Leishmania donovani *promastigotes J Cell Sci 2001 114 2461 2469 11559754 10.1242/jcs.114.13.2461

[B6] Petit PX Susin SA Zamzami N Mignotte B Kroemer G Mitochondria and programmed cell death: back to the future FEBS Lett 1996 396 7 13 10.1016/0014-5793(96)00988-X 8906857

[B7] Zhivotovsky B Kroemer G Apoptosis and genomic instability Nat Rev Mol Cell Biol 2004 5 752 762 10.1038/nrm1443 15340382

[B8] Degterev A Boyce M Yuan J A decade of caspases Oncogene 2003 22 8543 8567 10.1038/sj.onc.1207107 14634618

[B9] Madeo F Herker E Wissing S Jungwirth H Eisenberg T Fröhlich KU Apoptosis in yeast Curr Opin Microbiol 2004 7 655 660 10.1016/j.mib.2004.10.012 15556039

[B10] Madeo F Herker E Maldener C Wissing S Lächelt S Herlan M Fehr M Lauber K Sigrist SJ Wesselborg S A caspase-related protease regulates apoptosis in yeast Mol Cell 2002 9 911 917 10.1016/S1097-2765(02)00501-4 11983181

[B11] Uren A O'Rourke K Aravind LA Pisabarro MT Seshagiri S Koonin EV Dixit VM Identification of paracaspases and metacaspases: two ancient families of caspase-like proteins, one of which plays a key role in MALT lymphoma Mol Cell 2000 6 961 967 11090634 10.1016/s1097-2765(00)00094-0

[B12] Atkinson HJ Babbitt P Sajid M The global cysteine peptidase landscape in parasites Trends Parasitol 2009 25 573 581 10.1016/j.pt.2009.09.006 19854678 PMC3893884

[B13] Pollitt LC Colegrave N Khan SM Sajid M Reece SE Investigating the evolution of apoptosis in malaria parasites: the importance of ecology Parasites and vectors in press 21080937 10.1186/1756-3305-3-105 PMC3136143

[B14] Reece SE Drew D Gardner A Sex ratio adjustment and kin discrimination in malaria parasites Nature 2008 453 609 614 10.1038/nature06954 18509435 PMC3807728

[B15] Reece SE Ramiro RS Nussey DH Plastic parasites: sophisticated strategies for survival and reproduction? Evol Appl 2009 2 11 23 10.1111/j.1752-4571.2008.00060.x 20305703 PMC2836026

[B16] Reece SE Ali E Schneider P Babiker H Stress, drugs and the evolution of reproductive restraint in malaria parasites Proc Biol Sci 2010 277 3123 3129 10.1098/rspb.2010.0564 20484242 PMC2982055

[B17] Pollitt LC Mideo N Drew DR Schneider P Colegrave N Reece SE Competition and the Evolution of Reproductive Restraint in Malaria Parasites American Naturalist in press 21460544 10.1086/658175 PMC3939351

[B18] Nedelcu AM Driscoll WW Durand PM Herron MD Rashidi A On the paradigm of altruistic suicide in the unicellular world Evolution 2010 20722725 10.1111/j.1558-5646.2010.01103.x

[B19] Steller H Mechanisms and genes of cellular suicide Science 1995 267 1445 1449 10.1126/science.7878463 7878463

[B20] Wyllie AH Glucocorticoid-induced thymocyte apoptosis is associated with endogenous endonuclease activation Nature 1980 284 555 556 10.1038/284555a0 6245367

[B21] Newmeyer DD Farschon DM Reed JC Cell-free apoptosis in *Xenopus *egg extracts: inhibition by Bcl-2 and requirement for an organelle fraction enriched in mitochondria Cell 1994 79 353 364 10.1016/0092-8674(94)90203-8 7954801

[B22] Kroemer G Reed JC Mitochondrial control of cell death Nat Med 2000 6 513 519 10.1038/74994 10802706

[B23] Kluck RM Bossy-Wetzel E Green DR Newmeyer DD The release of cytochrome c from mitochondria: a primary site for Bcl-2 regulation of apoptosis Science 1997 275 1132 1136 10.1126/science.275.5303.1132 9027315

[B24] Susin SA Lorenzo HK Zamzami N Marzo I Snow BE Brothers GM Mangion J Jacotot E Costantini P Loeffler M Molecular characterization of mitochondrial apoptosis-inducing factor Nature 1999 397 441 446 10.1038/17135 9989411

[B25] Parrish J Li L Klotz K Ledwich D Wang X Xue D Mitochondrial endonuclease G is important for apoptosis in *C. elegans* Nature 2001 412 90 94 10.1038/35083608 11452313

[B26] Reape TJ McCabe PF Apoptotic-like regulation of programmed cell death in plants Apoptosis 2010 15 249 256 10.1007/s10495-009-0447-2 20094801

[B27] Sundström JF Vaculova A Smertenko AP Savenkov EI Golovko A Minina E Tiwari BS Rodriguez-Nieto S Zamyatnin AA Jr Välineva T Tudor staphylococcal nuclease is an evolutionarily conserved component of the programmed cell death degradome Nat Cell Biol 2009 11 1347 1354 10.1038/ncb1979 19820703

[B28] Yordanova ZP Iakimova ET Cristescu SM Harren FJ Kapchina-Toteva VM Woltering EJ Involvement of ethylene and nitric oxide in cell death in mastoparan-treated unicellular alga *Chlamydomonas reinhardtii* Cell Biol Int 2010 34 301 308 10.1042/CBI20090138 19947911

[B29] Sharon A Finkelstein A Shlezinger N Hatam I Fungal apoptosis: function, genes and gene function FEMS Microbiol Rev 2009 33 833 854 10.1111/j.1574-6976.2009.00180.x 19416362

[B30] Madeo F Fröhlich E Fröhlich KU A yeast mutant showing diagnostic markers of early and late apoptosis J Cell Biol 1997 139 729 734 10.1083/jcb.139.3.729 9348289 PMC2141703

[B31] Wissing S Ludovico P Herker E Büttner S Engelhardt SM Decker T Link A Proksch A Rodrigues F Corte-Real M An AIF orthologue regulates apoptosis in yeast J Cell Biol 2004 166 969 974 10.1083/jcb.200404138 15381687 PMC2172025

[B32] Oda K Kawasaki N Fukuyama M Ikeda S Ectopic expression of mitochondria endonuclease Pnu1p from *Schizosaccharomyces pombe *induces cell death of the yeast J Biochem Mol Biol 2007 40 1095 1099 18047809 10.5483/bmbrep.2007.40.6.1095

[B33] Bauer MK Schubert A Rocks O Grimm S Adenine nucleotide translocase-1, a component of the permeability transition pore, can dominantly induce apoptosis J Cell Biol 1999 147 1493 1502 10.1083/jcb.147.7.1493 10613907 PMC2174250

[B34] Mpoke S Wolfe J DNA digestion and chromatin condensation during nuclear death in *Tetrahymena* Exp Cell Res 1996 225 357 365 10.1006/excr.1996.0186 8660924

[B35] Ali M Al-Olayan EM Lewis S Matthews H Hurd H Naturally occurring triggers that induce apoptosis-like programmed cell death in *Plasmodium berghei *ookinetes PLoS One 2010 5 10.1371/journal.pone.0012634 PMC2936559 20844583

[B36] Picot S Burnod J Bracchi V Chumpitazi BF Ambroise-Thomas P Apoptosis related to chloroquine sensitivity of the human malaria parasite *Plasmodium falciparum* Trans R Soc Trop Med Hyg 1997 91 5 590 591 10.1016/S0035-9203(97)90039-0 9463676

[B37] Akematsu T Endoh H Role of apoptosis-inducing factor (AIF) in programmed nuclear death during conjugation in *Tetrahymena thermophila* BMC Cell Biol 2010 11 13 10.1186/1471-2121-11-13 20146827 PMC2829475

[B38] Gannavaram S Vedvyas C Debrabant A Conservation of the pro-apoptotic nuclease activity of endonuclease G in unicellular trypanosomatid parasites J Cell Sci 2008 121 99 109 10.1242/jcs.014050 18073240

[B39] Sen N Das BB Ganguly A Mukherjee T Bandyopadhyay S Majumder HK Camptothecin-induced imbalance in intracellular cation homeostasis regulates programmed cell death in unicellular hemoflagellate *Leishmania donovani* J Biol Chem 2004 279 52366 52375 10.1074/jbc.M406705200 15355995

[B40] Nedelcu AM Comparative genomics of phylogenetically diverse unicellular eukaryotes provide new insights into the genetic basis for the evolution of the programmed cell death machinery J Mol Evol 2009 68 256 268 10.1007/s00239-009-9201-1 19209377

[B41] Meslin B Barnadas C Boni V Latour C De Monbrison F Kaiser K Picot S Features of apoptosis in *Plasmodium falciparum *erythrocytic stage through a putative role of PfMCA1 metacaspase-like protein J Infect Dis 2007 195 1852 1859 10.1086/518253 17492602

[B42] The *MEROPS *peptidase database http://merops.sanger.ac.uk

[B43] Mottram JC Helms MJ Coombs GH Sajid M Clan CD cysteine peptidases of parasitic protozoa Trends Parasitol 2003 19 182 187 10.1016/S1471-4922(03)00038-2 12689649

[B44] Helms MJ Ambit A Appleton P Tetley L Coombs G Mottram JC Bloodstream form *Trypanosoma brucei *depend upon multiple metacaspases associated with RAB11-positive endosomes J Cell Sci 2006 119 1105 1138 10.1242/jcs.02809 16507595

[B45] Sajid M McKerrow J Cysteine proteases of parasitic organisms Mol Biochem Parasitol 2002 120 1 1 21 10.1016/S0166-6851(01)00438-8 11849701

[B46] Le Chat L Sinden R Dessens J The role of metacaspase 1 in Plasmodium berghei development and apoptosis Mol Biochem Parasitol 2007 153 1 41 47 10.1016/j.molbiopara.2007.01.016 17335919 PMC2075530

[B47] WU-BLAST - Protein Database http://www.ebi.ac.uk/Tools/sss/wublast/

[B48] Ito J Fukuda H ZEN1 is a key enzyme in the degradation of nuclear DNA during programmed cell death of tracheary elements Plant Cell 2002 14 3201 3211 10.1105/tpc.006411 12468737 PMC151212

[B49] Lyon CJ Evans CJ Bill BR Otsuka AJ Aguilera RJ The *C. elegans *apoptotic nuclease NUC-1 is related in sequence and activity to mammalian DNase II Gene 2000 252 147 154 10.1016/S0378-1119(00)00213-4 10903446

[B50] Sakahira H Enari M Nagata S Cleavage of CAD inhibitor in CAD activation and DNA degradation during apoptosis Nature 1998 391 96 99 10.1038/34214 9422513

[B51] Yokoyama H Mukae N Sakahira H Okawa K Iwamatsu A Nagata S A novel activation mechanism of caspase-activated DNase from *Drosophila melanogaster* J Biol Chem 2000 275 17 12978 12986 10.1074/jbc.275.17.12978 10777599

[B52] Durand P VDM Verlag Dr. Müller On the molecular evolution of the Plasmodium genome: Origin and Evolution of parasite genome 2010

[B53] Finn RD Mistry J Tate J Coggill P Heger A Pollington JE Gavin OL Gunasekaran P Ceric G Forslund K The Pfam protein families database Nucleic Acids Res 2010 39 Database D211 222 10.1093/nar/gkp985 19920124 PMC2808889

[B54] The Pfam database, a large collection of protein families, each represented by multiple sequence alignments and hidden Markov models (HMMs) http://pfam.sanger.ac.uk/

[B55] Candé C Cecconi F Dessen P Kroemer G Apoptosis-inducing factor (AIF): key to the conserved caspase-independent pathways of cell death? J Cell Sci 2002 115 Pt 24 4727 4734 10.1242/jcs.00210 12432061

[B56] Wang X Yang C Chai J Shi Y Xue D Mechanisms of AIF-mediated apoptotic DNA degradation in *Caenorhabditis elegans* Science 2002 298 1587 1592 10.1126/science.1076194 12446902

[B57] Arnoult D Tatischeff I Estaquier J Girard M Sureau F Tissier J Grodet A Dellinger M Traincard F Kahn A On the evolutionary conservation of the cell death pathway: mitochondrial release of an apoptosis-inducing factor during *Dictyostelium discoideum *cell death Mol Biol Cell 2001 12 3016 3030 11598188 10.1091/mbc.12.10.3016 PMC60152

[B58] Fontana L Partridge L Longo V Extending healthy life span--from yeast to humans Science 2010 328 321 326 10.1126/science.1172539 20395504 PMC3607354

[B59] Boyer M Wisniewski-Dyé F Cell-cell signalling in bacteria: not simply a matter of quorum FEMS Microbiol Ecol 2009 70 1 19 10.1111/j.1574-6941.2009.00745.x 19689448

[B60] Levine M Tjian R Transcription regulation and animal diversity Nature 2003 424 6945 147 151 10.1038/nature01763 12853946

[B61] Wu X Chi X Wang P Zheng D Ding R Li Y The evolutionary rate variation among genes of HOG-signaling pathway in yeast genomes Biol Direct 2010 5 46 10.1186/1745-6150-5-46 20618989 PMC2914728

[B62] Peisajovich SG Garbarino J Wei P Lim WA Rapid diversification of cell signaling phenotypes by modular domain recombination Science 2010 328 368 372 10.1126/science.1182376 20395511 PMC2975375

[B63] Dean S Marchetti R Kirk K Matthews KR A surface transporter family conveys the trypanosome differentiation signal Nature 2009 459 213 217 10.1038/nature07997 19444208 PMC2685892

[B64] Shiflett AM Johnson PJ Mitochondrion-related organelles in eukaryotic protists Annu Rev Microbiol 2010 64 409 429 10.1146/annurev.micro.62.081307.162826 20528687 PMC3208401

[B65] Chose O Noël C Gerbod D Brenner C Viscogliosi E Roseto A A form of cell death with some features resembling apoptosis in the amitochondrial unicellular organism Trichomonas vaginalis Exp Cell Res 2002 276 1 32 39 10.1006/excr.2002.5496 11978006

[B66] Chose O Sarde CO Gerbod D Viscogliosi E Roseto A Programmed cell death in parasitic protozoans that lack mitochondria Trends Parasitol 2003 19 12 559 564 10.1016/j.pt.2003.09.016 14642765

[B67] Kaczanowski S Siedlecki P Zielenkiewicz P The High Throughput Sequence Annotation Service (HT-SAS) - the shortcut from sequence to true Medline words BMC Bioinformatics 2009 10 148 10.1186/1471-2105-10-148 19445703 PMC2694793

[B68] High Throughput Sequence Annotation Service (HT-SAS) http://miron.ibb.waw.pl/htsas/10.1186/1471-2105-10-148 PMC2694793 19445703

